# Ethnic/racial minorities’ and migrants’ access to COVID-19 vaccines: A systematic review of barriers and facilitators

**DOI:** 10.1016/j.jmh.2022.100086

**Published:** 2022-02-18

**Authors:** Mohammed Abba-Aji, David Stuckler, Sandro Galea, Martin McKee

**Affiliations:** a Directorate of Public Health and Humanitarian Services, Medical Services Branch, Nigerian Air Force, Abuja, Nigeria; b Department of Social and Political Science, Bocconi University, Milan, Italy; c School of Public Health, Boston University, 715 Albany Street - Talbot 301, Boston, MA, United States; d Department of Health Services Research and Policy, London School of Hygiene and Tropical Medicine, 15-17 Tavistock Place, London, United Kingdom

**Keywords:** Migrants, Ethnic/racial minorities, Vaccine hesitancy, Vaccine uptake/coverage, Barriers/facilitators to vaccination

## Abstract

**Background:**

There are widespread concerns that ethnic minorities and migrants may have inadequate access to COVID-19 vaccines. . Improving vaccine uptake among these vulnerable groups is important towards controlling the spread of COVID-19 and reducing unnecessary mortality. Here we perform a systematic review of ethnic minorities’ and migrants’ access to and acceptance of COVID-19 vaccines.

**Methods:**

We searched PubMed and Web of Science databases for papers published between 1 January 2020 and 7 October 2021. Studies were included if they were peer-reviewed articles; written in English, included data or estimates of ethnic minorities’ or migrants’ access to vaccines; and employed either qualitative or quantitative methods. Of a total of 248 studies screened, 33 met these criteria and included in the final sample. Risk of bias in the included studies was assessed using Newcastle Ottawa Scale and Critical Appraisal Skills Program tools. We conducted a Synthesis Without Meta-analysis for quantitative studies and a Framework synthesis for qualitative studies.

**Results:**

31 of the included studies were conducted in high-income countries, including in the US (*n* = 17 studies), UK (*n* = 10), Qatar (*n* = 2), Israel (*n* = 1) and France (*n* = 1). One study was in an upper middle-income country -China (*n* = 1) and another covered multiple countries (*n* = 1). 26 studies reported outcomes for ethnic minorities while 9 studies reported on migrants. Most of the studies were quantitative -cross sectional studies (*n* = 24) and ecological (*n* = 4). The remaining were qualitative (*n* = 4) and mixed methods (*n* = 1). There was consistent evidence of elevated levels of COVID-19 vaccine hesitancy among Black/Afro-Caribbean groups in the US and UK, while studies of Hispanic/Latino populations in the US and Asian populations in the UK provided mixed pictures, with levels higher, lower, or the same as their White counterparts. Asians in the US had the highest COVID-19 vaccine acceptance compared to other ethnic groups. There was higher vaccine acceptance among migrant groups in Qatar and China than in the general population. However, migrants to the UK experienced barriers to vaccine access, mainly attributed to language and communication issues. Lack of confidence, mainly due to mistrust of government and health systems coupled with poor communication were the main barriers to uptake among Black ethnic minorities and migrants.

**Conclusions:**

Our study found that low confidence in COVID-19 vaccines among Black ethnic minorities driven by mistrust and safety concerns led to high vaccine hesitancy in this group. Such vaccine hesitancy rates constitute a major barrier to COVID-19 vaccine uptake among this ethnic minority. For migrants, convenience factors such as language barriers, fear of deportation and reduced physical access reduced access to COVID-19 vaccines. Building trust, reducing physical barriers and improving communication and transparency about vaccine development through healthcare workers, religious and community leaders can improve access and facilitate uptake of COVID-19 vaccines among ethnic minority and migrant communities.

## Introduction

The COVID-19 pandemic has had devastating consequences for health and the economy worldwide. There have been almost 5 million deaths, almost certainly an underestimate, by October 2021 (WHO Coronavirus, 2021) and virtually every country reported negative economic growth in 2020 (World Economic Outlook, 2020). The emergence of the Omicron variant and its related lineages is a reminder that the pandemic is not over, with continued disruptions likely through 2022 (World Economic Outlook Update, 2022). However, these aggregate figures conceal deep inequalities within populations, with widespread evidence that ethnic minorities and migrants have been disproportionately affected, both by the effects of infections and of countermeasures such as lockdowns (Hayward et al., 2021; Katikireddi et al., 2021). For example, a recent cross-sectional analysis in the United States found that, African American, Latino, and indigenous populations have been disproportionately affected by COVID-19-related infections and associated morbidity and mortality (Vahidy et al., 2020). Similarly, a systematic review which explored ethnicity and clinical outcomes reported that individuals of Black and Asian ethnicity in the UK were at increased risk of COVID-19 infection compared to White individuals (Sze et al., 2020). A recent systematic review on migrant populations from 15 high income countries reported that migrants had higher risk of exposure to, and infection with, COVID-19 than native populations (Hayward et al., 2021). However, despite the health and economic benefits that vaccines provide, migrants also have historically had low vaccine uptake compared to host populations (Tankwanchi et al., 2021). While the situation varies between countries and with different vaccines, this has been attributed, in varying degrees, to barriers to access and vaccine hesitancy.

There is now widespread agreement that vaccination against COVID-19 is critical to global emergence from the pandemic. It offers protection against transmission, protects against severe symptoms and reduces the risk of death in those infected with SARS CoV-2 (Vitiello et al., 2021; Mostaghimi et al., 2021; de et al., 2021). As of 13 December 2021, a total of 8,200,642,671 vaccine doses have been administered globally (WHO Coronavirus, 2021). However, there are concerns that just as these groups have been disproportionately harmed by higher infection rates and worse COVID-19 outcomes, they continue to be disproportionately harmed by lower vaccination uptake as vaccine programs gather pace, leaving them further behind as the world recovers. Here we report a systematic review of the evidence on whether this is happening and what might be done to overcome it.

The WHO SAGE Working Group on Vaccine Hesitancy defines vaccine hesitancy as “delay in acceptance or refusal of vaccination despite availability of vaccination services, a complex phenomenon and is often contextually bounded, influenced by factors such as complacency, convenience and confidence.” (MacDonald, 2015) However, whatever the reasons, the ability to control the pandemic in populations will ultimately depend on extending coverage to those least likely to receive vaccines.

We are unaware of any systematic review so far that has examined uptake of COVID-19 vaccines by ethnic minorities and migrants globally. We now seek to fill this gap by synthesizing the peer-reviewed literature on this issue, so as to provide an understanding of barriers and facilitators related to COVID-19 vaccine uptake in these groups with a view to identifying potential interventions. We do so by means of a systematic review and narrative synthesis of quantitative studies on COVID-19 vaccine access and acceptance among ethnic minority and migrant populations. Additionally, we conducted a framework synthesis, (Flemming and Noyes, 2022) using the best fit approach for qualitative studies that explored barriers and facilitators of COVID-19 vaccine access and acceptance among migrants and ethnic minority populations. We define ethnic minorities “as a group of people who differ in race or in national, religious, or cultural origin from the dominant group of the country of study” and as such may face discrimination and other barriers to accessing health services (Ethnic Minorities | Scholastic 2021). “Migrants” include refugees, asylum seekers and internally displaced persons and often face administrative, financial, legal, and language barriers to access the health system (World Health Organization, 2018).

## Methods

### Search strategy

We electronically searched PubMed and Web of Science databases covering the period from prior to the COVID outbreak, 1 Jan 2020, through to the time of the search, 7 October 2021, following PRISMA guidelines (Moher et al., 2009). The review was registered with PROSPERO (CRD42021278123) on 13 September 2021 (Abba-Aji et al., 2021).

The search included three main keywords: migrants, ethnic groups and COVID-19 vaccine. For migrants, we searched for existing systematic reviews and operationalized those keywords, including various permutations of asylum seekers, refugees, and internally displaced persons. For COVID-19 vaccines, a MeSH term has been created which we employed in our search. For ethnic groups, we recognize that this is an area that faces many terminological challenges because of the use of words that have particular meanings in different contexts or are in certain respects synonymous. Fortunately this has also been recognised by those developing the MESH term “Ethnic group”, which includes groups such as “African Americans, Amish, Arabs, Asian Americans, Hispanic Americans, Mexican Americans, Indigenous Peoples, Alaskan Natives, Jews, Roma”. In addition, the term “Black Americans” is also indexed under “African Americans” in Pubmed. For PubMed our search was as follows:

“migrants”[All Fields]OR “transients and migrants”[All Fields] OR “migrant”[All Fields] OR “migrants”[All Fields]) OR “Ethnic Groups”[MeSH Terms]) AND (“covid 19”[All Fields] OR “covid 19”[MeSH Terms] OR “covid 19 vaccines”[All Fields] OR “covid 19 vaccines”[MeSH Terms]

A full verbatim search strategy for PubMed and Web of Science is outlined in Appendix 1.

This initial search yielded 204 articles from Pubmed and 87 from Web of Science. Of these, 43 records were identified as duplicates through automated duplication detection by Covidence v2.0, (Covidence systematic review software 2022) and hand search, leaving a total of 248 records for further screening.

### Inclusion/exclusion criteria

We applied a series of inclusion and exclusion criteria during screening and eligibility stages. Articles were included if: i) they were peer-reviewed articles; ii) written in English; iii) they included data or estimates of migrants’ access to vaccines; and iv) they were a qualitative, quantitative or mixed method study.

We excluded papers that were not research studies or were reviews, such as commentaries, editorials, correspondences, systematic literature reviews and preprint articles. We further excluded population studies which did not report outcomes on either migrants or ethnic minority populations or when such outcomes could not be disaggregated from the general population. Papers were also excluded if they only described or proposed vaccine access policies without reporting on our outcome of interest. Finally we excluded studies on access or participation in COVID-19 vaccines trials and studies reporting on migrants and ethnic minority access to vaccines other than COVID-19 vaccines.

**Fig. 1:**
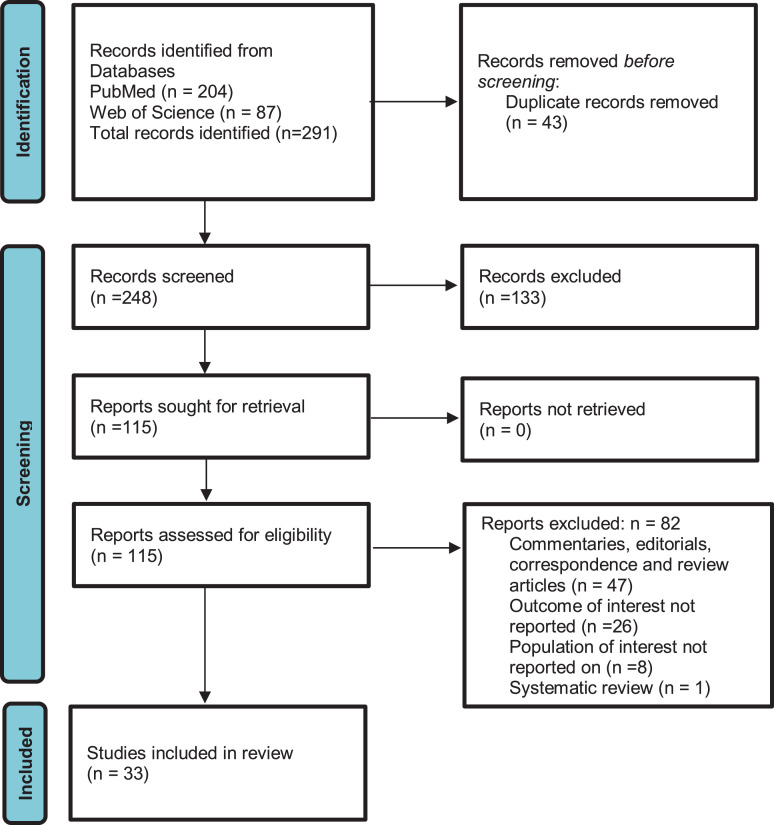
PRISMA Flow diagram.

Screening based on title and abstract was performed and reviewed independently by two authors (MA and DS). Any conflicts were resolved by consensus. The 2 reviewers screened titles and abstracts, removed duplicates and extracted data with Covidence 2.0 systematic review software. This excluded 133 studies, leaving 115 potentially eligible. Of these, 82 were excluded because they were not research studies (*n* = 47); did not report outcomes pertaining to vaccine access (*n* = 26); did not report data about migrants or ethnic minorities (*n* = 8); or was a systematic review (*n* = 1) (note: some articles had multiple reasons for exclusion; for brevity only the main one is included here). This left 33 articles in the final systematic review sample for data analysis and quality assessment Fig. 1. further describes the process of inclusion / exclusion.

### Data extraction, analysis and quality assessment

We extracted main study parameters into a summary Excel table. For quantitative studies this included: vaccine hesitancy scores, coverage rates/proportions, willingness to pay for and intention to receive COVID-19 vaccines. These outcomes were reported as odds ratios, relative risks, and proportions. For qualitative studies, factors that promoted or discouraged acceptance or access of COVID-19 vaccines among ethnic minority and migrant populations were extracted as the outcomes of interest.

Additionally we extracted data on the authors, country of study, study design, data collection method, sampling period, study population and type of outcome reported. For population studies we further extracted the migrants and ethnic minority sub group for which outcomes were reported, as well as any specific information on barriers/facilitators for accessing the COVID 19 vaccination.

For quantitative studies, quality was assessed using an adapted Newcastle-Ottawa scale based on study dimensions of sample selection, comparability and outcome ascertainment (Wells et al., 2021). Two reviewers (MA and DS) independently assessed the risk of bias and any uncertainty was resolved by contacting the third independent reviewer (MM). For qualitative studies, we used the Critical Appraisal Skills Program consisting of a 10-item questionnaire, such as clear aim of the research and appropriateness of qualitative methodology (Brice, 2021). We used both tools to assess the quality of a mixed-methods study.

Since there was considerable methodological and clinical heterogeneity across study populations, designs, and endpoints in the reviewed studies we did not perform a meta-analysis.

### Funding source

No ethics review was required as the study involved only secondary analysis of published studies. There was no direct funding source for this study.

## Results

Of the 33 studies included in the final sample, nearly all were conducted in high-income countries, including in the US (*n* = 17), UK (*n* = 10), Qatar (*n* = 2), Israel (*n* = 1) and France (*n* = 1). One study was in an upper middle-income country -China (*n* = 1) and another covered multiple countries (*n* = 1). No study covered populations in low- or lower-middle income countries. Most of the studies were quantitative -cross sectional studies (*n* = 24) and ecological (*n* = 4). The remaining were qualitative (*n* = 4) and mixed methods (*n* = 1) Table 1. provides a description of included studies with a summary of their key findings.

**Table 1:** Summary of quantitative studies.

Author and Year	Country	Study design	Study population and sample size	Sub-group	Outcome(s) reported	Summary of findings in ethnic minorities/migrants	NOS
Han et al., 2021	China	Cross-sectional	migrants in Shanghai(*N* = 2126)	N/A	Acceptance and willingness to pay for COVID-19 vaccine	89.1% acceptance; Median WTP USD 46; Perceived susceptibility and confidence in vaccines was associated with higher acceptance and Willingness to pay.	8
Longchamps et al., 2021	France	Cross-sectional	Residents of homeless shelters(*N* = 235)	Migrants	Intention to be vaccinated	Legal residents more hesitant than non legal residents	5
Green et al., 2021	Israel	Cross-sectional	General population(*N* = 957)	Ethnic minorities	Willingness to receive vaccines.	Prevalence rates of those who would refuse the vaccine at any stage were almost always higher among Arabs than Jews in both sexes.The most outstanding ethnic difference was in the total refusal of the vaccine, where the Arab participants were much more likely to say they would refuse vaccine than the Jewish participants.	7
Qunaibi et al., 2021	Multi-country	Cross-sectional	Arab population (*N* = 36,220 )	Migrants	Willingness to receive vaccines(dichotomized to vaccine acceptance and hesitancy)	Participants in the Arab World slightly more VH than those living outside (83.3% vs. 81.2%) Those living in North America were the least hesitant (76.3%), while those living in Turkey had the highest hesitancy (83.6%).	4
Khaled et al., 2021	Qatar	Cross-sectional	Adult population (*N* = 670)	Migrants	Vaccine willingness(categorized into vaccine accepting, hesitant and refusing)	Migrants were less VH than Qataris	7
Alabdulla et al., 2021	Qatar	Cross-sectional	General population(*N* = 7821)	Migrants	Intention to accept vaccine	Overall vaccine hesitancy among the local Qataris of working age was 42.57% compared to 16.71% for the immigrant population.	7
Williams et al., 2021	UK	Cross-sectional	Scottish adult population(*N* = 3436)	Ethnic minorities	Intention to be vaccinated (Vaccine Hesitant and willing)	After adjusting for other variables such as age, income and education, BAME groups had 3x lower levels of intention than those of white ethnicity.	5
Gibbon et al., 2021	UK	Cross-sectional	Psychiatric in-patients(*N* = 92)	Ethnic minorities	Uptake of vaccine	BAME background were more likely to decline the vaccine than White British patients.	4
Robertson et al., 2021	UK	Cross-sectional	General population(*N* = 957)	Ethnic minorities and migrants	Vaccine hesitancy	Odds ratios for vaccine hesitancy were 13.42 (95% CI:6.86, 26.24) in Black and 2.54 (95% CI:1.19, 5.44) in Pakistani/Bangladeshi groups (compared to White British/Irish); Asian background had higher acceptance.Migrants did not have greater odds of vaccine hesitancy (OR 0.99 95% CI: 0.67, 1.48)Black or Black British ‘Don't trust vaccines’ (29.2% vs 5.7%) and the Pakistani or Bangladeshi have expressed higher concerns about side-effects (35.4% vs 8.6%).	7
Paul et al., 2021	UK	Cross-sectional	General population(*N* = 32,361)	Ethnic minorities	Vaccine attitudesIntention to receive COVID-19 vaccine	Higher mistrust among ethnic minoritiesNo significant difference in intention to vaccinate among ethnic minorities.	8
Bell et al., 2020	UK, England	Mixed methods	Parents and guardians(*N* = 1252)	Ethnic minorities	Vaccine acceptance(dichotomized into likely to accept and likely to reject)	Black, Asian, Chinese, Mixed or Other ethnicity 2.7 times (95%CI: 1.27–5.87) more likely to reject for themselves and their children than White participants.	
Sethi et al., 2021	UK	Cross-sectional	Adult population (*N* = 4884)	Ethnic minorities	Vaccine acceptance	This contrasts with other studies, BAME community (OR=5.48) were more likely to take an approved vaccine. mean scores of the BAME significantly higher than that of the Non BAME, although SDs were lower. Variation of the scores of the BAME community was higher than the non-BAME community. A possible indication that perception of vaccines differs widely across the BAME community.	6
Kelly et al., 2021	US	Cross-sectional	Adult population(*N* = 2279)	Ethnic minorities	Willingness to receive a vaccine(dichotomized as willing vs. not willing)	Black respondents were less willing to get the vaccine than White respondents (53% vs. 79%, OR = 0.34, 95% CI = 0.22–0.54, Hispanics more willing than White respondents (80% vs. 75%, *p* < 0.001).	6
Famuyiro et al., 2022	US	Cross-sectional	Healthcare workers(*N* = 300)	Ethnic minorities	Acceptance of vaccine (vaccine uptake readiness categorized as Yes, Later or No)	Asians 78.1%; Whites 71.2%; Hispanic 45.6%; Blacks 37.5%Blacks (OR=0.066, *p* = 0.010), and Hispanics (OR=0.11, *p* = 0.037) were less likely to accept the vaccineOn adjusting for perceived risk, sex, race/ethnicity, and age, only non-Hispanic Black remained statistically significant (adjusted OR=0.07, 95% confidence interval (CI), 0.01–0.59).	5
Thompson et al., 2021	US	Cross-sectional	Adult residents of Michigan.(*N* = 1835)	Ethnic minorities	Vaccine acceptance(Dichotomized Rejecting and accepting);Mistrust scores	Asian 64%; White 57%;Hispanics 42%;Multiracial or other 43%;; MENA 38%; Black 28%Black participants had the highest mistrust scores (mean [SD] score, 2.35 [0.96]).There was greater rejection among Black participants (*B* [SE], 0.51 [0.08]; *P* < .001) and less rejection among Asian (*B* [SE], −0.63 [0.14]; *P* < .001) and White (*B* [SE], −0.20 [0.07]; *P* = .005) participants.	7
Scott et al., 2021	US	Cross-sectional	Amish families(*N* = 1000)	N/A	Likelihood of accepting vaccine.Reasons for refusal/acceptance	75.7% did not intend to have their children receive a COVID-19 vaccine if one became available.Concern for adverse events more than religious reason;significantly more likely to recognize their doctor or nurse as the most influential people when making vaccines decisions.	4
N Williams et al., 2021	US	Ecological	Brooklyn, New York residents 2 604 747 residents	Ethnic minorities	Access to vaccination sites	Disparities in vaccination site access. Of note, district 16 had the highest percentage of the population below the poverty threshold (29.4%) and had no vaccination sites.The median population density per site among districts with lower poverty was 6793.6 persons per square mile per site, compared with a ratio nearly double of 11 263.4 persons per square mile per site among districts with higher poverty.	N/A
Olanipekun et al., 2021	US	Cross-sectional	COVID 19 Recovered African Americans patients (*N* = 119)	N/A	Vaccine acceptance; Factors for refusal	Overall, 30% responded they would accept a vaccine COVID-19 vaccine, 54% responded they would not, while 16% were undecided.Major reasons were combination of distrust in the vaccine efficacy irrespective of what the research shows and distrust of the pharmaceutical companies that produce vaccines (78%), fear of vaccination side effects (65%), and perceived immunity against COVID-19 re-infection (29%).	4
Viswanath et al., 2021	US	Cross-sectional	Adult population(*N* = 1012)	Ethnic minorities	Intention to vaccinate (dichotomized as likely and unlikely)	White 71.8; Hispanic 64.9%; Other ethnicities 72.4%;Black 51.8%.Among racial and ethnic groups, non-Hispanic Blacks are least likely to agree to vaccinate self or people in their care.No significant difference between whites, Hispanic and other ethnicities.	6
Bogart et al. 2021	US	Cross-sectional	HIV-positive Black Americans.(*N* = 101)	N/A	COVID-19 vaccine hesitancy.	54% Vaccine hesitancy among participants of the study.About 30% said they would not get vaccinated or treated.COVID-19 mistrust was related to greater vaccine hesitancy ; participants had greater trust in health care providers than the government.	6
Allen et al., 2021	US	Cross-sectional	Women(*N* = 396)	Ethnic minorities	Vaccine intention.	Chinese 70.7%; White 62.4%; Hispanic 53.5%;Multiracial or other 64.3%;Black 39.2%.After adjusting for socio-demographic, COVID-19-specific covariates, and trust in information about vaccination from healthcare professionals, Non-Latin Black women were significantly less likely to report that they would be vaccinated than Non-Latin White women.	7
Painter et al., 2021	US	Ecological	Adults12,537,841 vaccine recipients	Ethnic minorities	Vaccine coverage.	White 60.4%; multiple or other race/ethnicity,14.4% ; Hispanic/Latino 11.5%, ; Asian 6.0%,; Black 5.4%,; American Indian/Alaska Native; 2.0%.	N/A
Gatwood et al., 2021	US	Cross-sectional	Adult residents Tennessee (*N* = 1000)	Ethnic minorities	Likelihood of COVID-19 vaccination(dichotomized into Accepting and hesitant)	Black Americans were less likely to seek COVID-19 vaccination compared to Whites (AOR, 1.56; 95% CI, 1.002–2.427).	7
Wong et al., 2021	US	Ecological	Residents of North Carolina*N* = 2815,774.	Ethnic minorities	Vaccine administrationStrategies used to increase coverage	Proportion of vaccines administered to Black persons increased from 9.2% (95% CI = 9.1%–9.4%) to 18.7% (95% CI = 18.6%–18.9%) (*p*<0.001); during the same period; proportion administered to Hispanic persons increased from 3.9% (95% CI = 3.8%–4.0%) to 9.9% (95% CI = 9.8%–10.0%) (*p* < 0.001).Mapping, promoting shared accountability with providers for equitable vaccine distribution through public dashboards and individualized performance reporting, and building partnerships to support vaccine access.	N/A
Phillips et al., 2021	US	Cross-sectional	Adult Sexual and Gender Minorities.(*N* = 932)	Ethnic minorities	Interest in COVID-19 vaccine	Latino individuals were significantly less likely to be interested in a future COVID-19 vaccination (OR 0.40; 95% CI: 0.21–0.74).	4
Pingali et al., 2021	US	Ecological	Adult population (*N* = 9.6 million)	Ethnic minorities	Vaccine coverageCoverage with ≥1 COVID-19 vaccine dose	Asian (57.4%) ; White persons (54.6%) ; Hispanic (41.1%); Black persons (40.7%).	N/A
Malik et al., 2020	US	Cross-sectional	Adult population.(*N* = 672)	Ethnic minorities	Acceptance of COVID-19 vaccine	White 68% ;Asians 81%; Hispanics 68%.; American Indian/Alaska Native 74%; Blacks 40%.	6
Kuhn et al, 2021	US	Cross-sectional	Homeless people in Los Angeles (*N* = 90)	Ethnic minorities	Vaccine uptake/rejection	No significant differences in vaccine hesitancy across key demographic variables, including race.	5

26 studies reported outcomes for ethnic minorities of which only 7 focused primarily on this group. The remaining 19 focused on the general population or healthcare workers but additionally reported outcomes for ethnic minorities. The most frequently studied ethnic group was Blacks (*n* = 24) followed by Asians (*n* = 14), non-white Latin ethnic group (*n* = 12) and other ethnic minorities (*n* = 9).

**Fig. 2:**
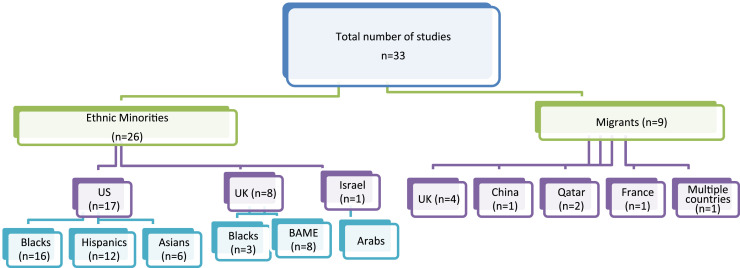
Summary of included studies by region and population.

9 studies reported outcomes for migrant populations. None of the studies reported outcomes for internally displaced persons. Sample sizes of included studies ranged considerably, from *n* = 90 (Kuhn et al., 2021) to *n* = 36,220 (Qunaibi et al., 2021). Fig. 2. summarises the studies by region and population.

### Vaccine access and acceptance/hesitancy in ethnic minorities *(n* *=* *26)*

First we summarize evidence on COVID-19 vaccine access and acceptance/hesitancy among ethnic minorities by country, starting with the US, and then move to those studies of migrant populations.

#### United States (*n* = 17)

We first provide comparative summaries on access and acceptance among minority ethnic groups. Next we summarize evidence about African Americans/Blacks (*n* = 16), followed by Hispanic/Latinx (*n* = 12), Asian (*n* = 6) and Amish groups. Several studies reported on multiple ethnic groups, individually or in aggregate.

Turning first to the comparative summaries, it appeared that Asians had the highest vaccine acceptance and lowest hesitancy rates, followed by White persons, then Hispanic and lastly African-Americans. Malik and colleagues reported intention rates of 81% in Asians, 68% in White populations, and 40% in African-Americans (Malik et al., 2020). Similar patterns were seen in a number of other studies at later time points. These inequalities persisted not just in intentions to be vaccinated per se but also in uptake. As one example, Pingali and colleagues investigated racial disparities in vaccination uptake from 14 December 2020 to May 2021, finding that, at the time, 57.4% of Asian populations, 54.6% of White populations, 41.1% of Hispanic populations, and 40.7% of African-Americans had received at least one COVID-19 vaccine dose (Pingali, 2021).

All but one of the US studies on ethnic minorities reported outcomes for Black ethnicity (*n* = 16). Almost all reported that Black groups had substantially lower vaccine uptake/acceptance, compared to their non-Latin white counterparts. In a national cross-sectional survey of adults, Black respondents were significantly less likely to accept the vaccine than White respondents (53% vs. 79%, OR = 0.34, 95% CI = 0.22–0.54 (Kelly et al., 2021). Viswanath and colleagues reported a similar gap (51.8% vs. 72.4%) (Viswanath et al., 2021). A later national cross-sectional survey by Malik and colleagues found lower rates in both groups (40% vs. 68%) (Malik et al., 2020). Similarly, in a survey among women in the US, Black women had significantly lower acceptance rates compared to White women (39.2% vs. 62.4%) (Allen et al., 2021). A survey of Tennessee adults reported that Black Americans in the state were less likely to accept the vaccines compared to their White counterparts (AOR, 1.56; 95% CI, 1.002–2.427) (Gatwood et al., 2021). The lowest intention to be vaccinated against COVID-19 was found among Black adults in Michigan where only 23% of this ethnic group expressed such an intention (compared to 57% of Whites) (Thompson et al., 2021). A study by Olanipekun and colleagues that looked at the attitudes of African Americans who had recovered from COVID-19 reported that only 30% of participants would accept a COVID-19 vaccine, with 54% declining and 16% undecided (Olanipekun et al., 2021). A hospital-based study by Bogart and colleagues assessed vaccine hesitancy among 101 HIV-positive Black Americans (Bogart et al., 2021). More than half of participants (54%) reported hesitancy about accepting a future COVID-19 vaccine and about a third said they would not get vaccinated. Only 2 studies reported no significant difference in vaccine acceptance between Blacks and Whites in the US. These were by Kuhn and colleagues who assessed uptake among homeless people in Los Angeles and by Philips and colleagues, who assessed vaccine acceptance by people from sexual and gender minorities (Kuhn et al., 2021; Phillips et al., 2021). In summary, there is strong evidence of higher rates of vaccine hesitancy among Black compared to White Americans with exceptions to this trend coming from studies investigating populations that are marginalized for other reasons. These differences persist even when adjusted for socio-demographic factors such as age, income and education (Gatwood et al., 2021).

Some studies have sought explanations for these differences. Famuyiro and colleagues hypothesized that greater health literacy and higher exposure to infection among healthcare workers might narrow inequalities in vaccine acceptance (Famuyiro et al., 2022). They conducted a cross-sectional survey in December 2020 among clinical and non-clinical community health workers in Texas offered Pfizer-BioNTech or Moderna vaccines (Scott et al., 2021). However, even in this population they found much lower acceptance among Black health workers (36%) than their Asian (78%) and White (71%) counterparts. This remained statistically significant (adjusted OR=0.07, 95% confidence interval (CI), 0.01–0.59) even after adjusting for perceived risk, sex and age.

12 of the 17 US studies reported data on Hispanic groups. Here, COVID-19 vaccine acceptance varied considerably. Some reported lower acceptance than among Whites (Thompson et al., 2021; Phillips et al., 2021, Famuyiro et al., 2022) while others found no significant difference (Kelly et al., 2021; Viswanath et al., 2021). In Famuyiro and colleagues’ study of health workers in Texas, Hispanics were less likely to accept the vaccine compared to their White colleagues (45.6% vs. 71.2% OR = 0.11, *p* = 0.037). A study by Thompson and colleagues on vaccine acceptance among residents of Michigan reported lower acceptance among Hispanics than Whites (42% vs. 57%) (Thompson et al., 2021). Allen et al. also reported that Hispanic women had lower acceptance than their White counterparts (53.5% vs. 62.4%) (Allen et al., 2021). Finally, a study among sexual and gender minorities reported that Latinx individuals were significantly less likely to be interested in accepting a future COVID-19 vaccination than Whites (OR 0.40; 95% CI: 0.21–0.74) (Phillips et al., 2021). A national survey on US adults conducted by Viswanath et al. and by Malik et al. found no significant difference in intention to vaccinate between Whites and Hispanics (Malik et al., 2020; Viswanath et al., 2021). Only one study found higher acceptance among Hispanics, a survey of the US adult population by Kelly and colleagues (Kelly et al., 2021). However the difference was small, at 80% among Hispanics and 75% among Whites (p < 0.001). In summary, the picture with this ethnic group is inconsistent and differences likely reflect other factors related to the populations sampled.

Finally, 6 of the 17 US studies on ethnic minorities examined hesitancy among Asian groups and multiracial/other ethnicities. Asians had the highest COVID-19 vaccine acceptance across all ethnic groups, ranging from 64% in a survey of Michigan residents to 81% in a US nationally representative survey (Malik et al., 2020; Thompson et al., 2021). Respondents categorized as multiracial/other ethnicities had acceptance patterns that were fairly similar to those of Whites. Acceptance in this group ranged from 43% in the survey of Michigan residents to 72.4% in a nationally representative survey (Viswanath et al., 2021). A cross sectional survey by Scott et al. assessed COVID-19 vaccination patterns of Amish families in northeast Ohio, finding that 75.7% of Amish parents did not intend to vaccinate their children with a COVID-19 vaccine (Scott et al., 2021).

#### *United Kingdom (n = 8)*

Of the 8 UK studies that reported outcomes for ethnic minorities, 5 reported their findings under a collective label, “BAME” (Black, Asian and Minority Ethnic Backgrounds) and only 3 differentiated particular ethnic groups (Williams et al., 2021; Gibbon et al., 2021; Robertson et al., 2021; Paul et al., 2020; Bell et al., 2020; Sethi et al., 2021; Freeman et al., 2020).

6 out of 8 studies reported lower vaccine acceptance proportions/ ratios among Black/BAME minorities compared to their White counterparts. For example, Robertson et al. reported a very high odds ratio for vaccine hesitancy of 13.42 (95% CI:6.86, 26.24) in Blacks compared to Whites (Robertson et al., 2021). Similarly, William et al., found that even after adjustment for socio-demographic factors, BAME groups still had 3 times lower levels of intention to vaccinate than those of white ethnicity (Williams et al., 2021). Paul et al., used a large survey to seek factors that predicted negative attitudes towards vaccines in general and intent to vaccinate against COVID-19 in particular (Paul et al., 2021). Although they found more frequent negative attitudes among ethnic minorities, these attitudes did not influence intentions by these groups to accept COVID-19 vaccines. Their findings were not reported for different ethnic groups, so caution is needed when drawing conclusions given the potential for heterogeneity. In contrast, Sethi et al. 2021 found that the BAME community were more likely to take an approved vaccine than Whites (OR = 5.48), a finding that the authors attribute to the disproportionate COVID-19 mortality among the BAME population (Sethi et al., 2021). However, it should be noted that this study too did not disaggregate its findings according to different ethnic groups (Blacks, Asians and Mixed ethnicity), although the authors did suggest that there may be variations.

#### *Israel (n = 1)*

One study reported on acceptance of vaccines by Arab Israelis in a study of ethnic and socio-demographic factors associated with attitudes towards COVID-19 vaccines. The authors reported significantly higher rejection by Arabs (29.9%) compared to Jews (7.7%) (*p* < 0.0001). Although women in general showed more hesitancy than men, this was significantly higher in Arab (41.0%) than Jewish women (17.2%) (Green et al., 2021). The authors suggest that the low willingness among Arab women could be related to a lack of confidence in COVID-19 vaccines, reflecting disinformation around the idea that the vaccines lead to infertility.

### Vaccine acceptance/hesitancy in migrants

Next we summarize evidence from the 9 studies that examined vaccine access and acceptance among migrants. These were from the UK (*n* = 4), Qatar (*n* = 2), France (*n* = 1), China (*n* = 1) and multiple countries (*n* = 1) (Qunaibi et al., 2021; Robertson et al., 2021; Han et al., 2021; Khaled et al., 2021; Alabdulla et al., 2021; Longchamps et al., 2021; Deal et al., 2021; Knights et al., 2021, Woodhead et al., 2022). None were from the US.

In a study of people living in homeless shelters across France, Longchamps et al. found higher COVID-19 vaccine hesitancy among legal residents (48.9%) than residents lacking legal status (35.9%) (Longchamps et al., 2021). A longitudinal survey in the UK population by Robertson et al. reported no difference in vaccine hesitancy between those born outside (OR 0.99 95% CI: 0.67–1.48) or within the UK (Robertson et al., 2021). Two studies conducted in Qatar both found higher acceptance of COVID-19 vaccines among migrants compared to Qatari nationals (Khaled et al., 2021; Alabdulla et al., 2021). In a national cross-sectional survey, where migrants comprised the majority, Alabdulla et al. reported vaccine hesitancy among Qataris of working age as 42.57% compared to 16.71% in the immigrant population (Alabdulla et al., 2021). Khaled et al. reported significantly lower vaccine hesitancy among white collar migrants (RRR = 0.32; 95% CI: 0.16–0.67) and blue collar migrants (RRR = 0.32; 95% CI: 0.12–0.87) compared to Qatari nationals (Khaled et al., 2021). Similarly, a study on migrant workers in China by Han et al. found a high willingness to accept the COVID-19 vaccines (89.1%) among this group (Han et al., 2021). However, a multinational cross-sectional study by Qunaibi et al. on COVID-19 vaccine hesitancy among Arab populations reported high COVID-19 vaccine hesitancy among surveyed participants living in the US (76.3%) and Turkey (81%) (Qunaibi et al., 2021). Overall, 5 out of the 6 studies that reported vaccine acceptance among migrants in different countries showed that they were largely willing to accept COVID-19 vaccines.

### Barriers to COVID-19 vaccine uptake

Taken together the studies revealed a number of barriers to COVID-19 vaccine uptake. We synthesize them using the 3Cs Model of Vaccine Hesitancy namely; confidence, convenience and complacency. Confidence was the most frequently reported barrier (*n* = 12), followed by convenience (*n* = 4) and complacency (*n* = 2). Some studies reported more than one barrier.

#### Confidence barriers

We will first present synthesized evidence of confidence barriers. The main drivers were mistrust in the healthcare system/government (*n* = 9) and vaccine safety concerns (*n* = 7).

One important qualitative study by Jimenez et al. provided potential reasons for mistrust among ethnic minorities in the US (Jimenez et al., 2021). The researchers sought to understand life experiences of ethnic minorities and how it affected their perspectives on COVID-19 and vaccine acceptance. They conducted group and individual interviews, including with 68 African-Americans recruited from New Jersey counties severely affected by the pandemic. They found that the devastating effects of the pandemic did not translate into COVID-19 vaccine acceptance, with participants expressing a high level of distrust of the vaccine. Specifically, they did not trust the vaccine development process, expressing concerns that the process had been “rushed” and that the vaccines might have long-term adverse effects. They also expressed mistrust of the healthcare system and government, voicing fears that they might be unwilling subjects of experiments.

As in the US, mistrust was a key barrier to vaccine acceptance among Afro-Caribbeans in the UK (Robertson et al., 2021; Paul et al., 2021). Even among those who were healthcare workers, suspicion and mistrust in the vaccine development process was especially salient, often reflecting concerns about poor and unethical past research.

Only a few investigated the role of mistrust in fueling hesitancy quantitatively. Thompson et al., in a study of adults in Michigan that examined the association between race/ethnicity and rejection of COVID-19 vaccine asked whether medical mistrust was a potential mediator, finding evidence that it was (Thompson et al., 2021). However, in a study among US women, non-Latin Black women remained less likely to accept COVID-19 vaccines even after adjusting for trust in information about vaccination from healthcare professionals (Allen et al., 2021).

#### Convenience barriers

Next we turn to convenience barriers. The WHO SAGE working group on vaccine hesitancy has categorized factors such as physical unavailability, unaffordability and unwillingness-to-pay, geographical inaccessibility and inability to understand (language and health literacy) as convenience barriers. Of these, inability to understand (language and health literacy) (*n* = 3) was the most commonly cited followed by geographical inaccessibility (*n* = 2) and unaffordability (*n* = 1).

Two qualitative studies in the UK conducted in person interviews with different categories of migrants in order to gain a deeper understanding on their perspectives on COVID-19 vaccine access (Deal et al., 2021; Knights et al., 2021). In the first study, Knights and colleagues found that the digitization of healthcare services coupled with language barriers reduced migrants’ access to information on healthcare services. This exacerbated existing healthcare inequalities, and especially access to COVID-19 vaccines (Knights et al., 2021). Additionally, the study reported that undocumented migrants expressed fears that they might be targeted for deportation when they present for COVID-19 vaccinations. In the second study, Deal et al. identified costs associated with the vaccine, both direct (in this case a perceived cost) and indirect (e.g. travel), as a major barrier to vaccine uptake among some migrants (Deal et al., 2021). The fact that these concerns emerged despite the UK government stating that neither identification nor payment was required at the point of vaccination points to a problem with information dissemination to migrants.

Several studies speculated that convenience barriers, particularly geographical inaccessibility (e.g. long travel distances, long queues and wait times) could have driven low COVID-19 vaccine uptake among ethnic minorities and migrants. However, only a few investigated their importance quantitatively. One was by William and colleagues, undertaken in Brooklyn, New York City, where 52% of the population identified as Latin or Black (Williams et al., 2021). They found fewer vaccination sites (median 4; range 0–5) among districts with less White (non-Hispanic) race/ethnicity and more (median 6; range 3–8) among districts with greater White (non-Hispanic) race/ethnicity. More worrisome was the fact that Brooklyn's district 16, a largely minority district with the highest percentage of the population below the poverty threshold, had no vaccination sites.

#### Barriers due to complacency

Only 2 studies reported complacency as a barrier to vaccine uptake among ethnic minorities and migrants. Olanipekun and colleagues assessed intentions to vaccinate among COVID-19 recovered African Americans, finding that 54% of study participants were unwilling to accept vaccines (Olanipekun et al., 2021). Of those unwilling, 29% cited “perceived immunity against COVID-19 re-infection” as their reason for refusal. In the study by Knights and colleagues, migrants with BAME ethnicity believed COVID-19 was a “European infection” and that therefore they (migrants) were immune to it (Knights et al., 2021).

### Facilitators to COVID-19 vaccines uptake

Some strategies to facilitate better access and increase acceptance to COVID-19 vaccines for migrants and ethnic minorities were highlighted. These revolved around building trust, improving communication with ethnic minorities and migrant communities and improving physical access to COVID-19 vaccines.

#### Building trust

Most studies reported improving confidence as facilitator to COVID-19 vaccine uptake, involving building trust between health institutions and ethnic minority and migrant populations. This featured prominently in all 4 qualitative studies (Deal et al., 2021; Knights et al., 2021; Woodhead et al., 2022; Jimenez et al., 2021). Despite some evidence of medical mistrust, some studies reported how healthcare workers, along with community/religious leaders were the most trusted sources of COVID-19 vaccine information for ethnic minorities and migrants (Bogart et al., 2021; Phillips et al., 2021). This was also true in the Amish population who were significantly more likely to recognize their doctor or nurse as the most influential people when making vaccination decisions (Scott et al., 2021).

#### Improving communication

There was consensus that more effective communication by health bodies is needed to facilitate COVID-19 vaccine uptake in ethnic minority/racial and migrant groups. Contextual factors, reflecting historical, environmental and health system factors have weakened migrants’ trust in COVID-19 vaccines and ultimately their decision to accept/reject the vaccines. It was suggested that these were best addressed through influential leaders in the communities concerned, who could champion vaccine uptake within their local communities (Deal et al., 2021; Knights et al., 2021; Jimenez et al., 2021).

#### Improving physical access to vaccines

There is evidence that improving physical access to vaccination sites also improves uptake among ethnic minorities. Authors Wong et al. for example, found a substantial increase in COVID-19 uptake among ethnic minorities in North Carolina when access to vaccines was improved (Wong et al., 2021). Specifically, the proportion of Blacks and Hispanics vaccinated nearly doubled, almost completely closing the ethnic gap in the state population among those aged 16 years and above, when a combination of strategies to improve access had been used. These strategies relied on data mapping techniques to ensure that adequate doses were allocated to vaccine providers in close proximity to Black and Hispanic populations and forming partnerships between vaccine providers and faith/community-based organizations.

### Quality appraisal

Table 2 presents the results of the quality appraisal for cross-sectional studies. We assessed the cross-sectional studies (*n* = 24) using an adapted NOS protocol as outlined in the Methods section. Most studies were of moderate quality *n* = 22. The average score was approximately 6 stars (range = 4–8 stars). The most common quality issues were failures to justify sample size and to describe the response rate or the characteristics of the responders and the non-responders.

**Table 2:** Quality appraisal of cross-sectional studies (n-24) using Newcastle-Ottawa Scale.

**Author and Year**	**Selection: (Max. 3 stars)**			**Comparability (Max. 2 stars)**			**Outcome (Max. 5 stars)**		**Total score**
	**Representativeness of the sample**	**Sample size**	**Non respondents**	Presence of control/comparator group. *	Controls for any additional factor. *	**Ascertainment of outcome**	**Assessment of the outcome**	**Statistical test**	
Han 2021	*	*		*	*	**	*	*	**8**
Paul 2021	*	*		*	*	**	*	*	**8**
Alabdulla 2021	*			*	*	**	*	*	**7**
Allen 2021	*	*		*	*	*	*	*	**7**
Gatwood 2021	*	*		*		**	*	*	**7**
Green 2021	*	*		*	*	*	*	*	**7**
Khaled 2021	*	*	*	*		*	*	*	**7**
Robertson 2021	*	*		*	*	*	*	*	**7**
Thompson 2021		*		*	*	**	*	*	**7**
Freeman 2020	*	*		*		**	*	*	**7**
Bogart 2021	*			*		*	*	*	**6**
Kelly 2021	*	*		*		*	*	*	**6**
Viswanath 2021	*	*		*		*	*	*	**6**
Sethi 2021	*	*		*		*	*	*	**6**
Malik 2020	*	*		*		*	*	*	**6**
Famuyiro 2021				*		*	*	*	**5**
Longchamps 2021				*		**	*	*	**5**
Williams 2021			*	*		*	*	*	**5**
Kuhn 2021	*			*		*	**		**5**
Olanipekun 2021						**	*	*	**4**
Philips 2021				*		*	*	*	**4**
Qunaibi 2021				*		*	*	*	**4**
Scott 2021	*					*	*	*	**4**
Gibbon 2021				*		*	**		**4**

Table 3 presents the results of quality assessment for the qualitative studies. We assessed the studies using the CASP checklist . The checklist had 10 questions, each of which was given an answer, ‘Yes’, or ‘No’, or ‘Cannot tell’. As suggested by CASP, we did not create a summary score of the appraised studies. We retain articles of seemingly poor quality but report our assessment of potential biases.

**Table 3:** Quality appraisal of qualitative studies using CASP tool checklist.

**Authors and year**	**Q1**	**Q2**	**Q3**	**Q4**	**Q5**	**Q6**	**Q7**	**Q8**	**Q9**	**Q10**
**1. Knights et al., 2021**	**Y**	**Y**	**Y**	**Y**	**Y**	**Y**	**Y**	**?**	**Y**	**Y**
**2. Jimanez et al., 2021**	**Y**	**Y**	**Y**	**Y**	**Y**	**N**	**Y**	**?**	**Y**	**Y**
**3. Deal et al., 2021**	**Y**	**Y**	**Y**	**Y**	**Y**	**N**	**Y**	**Y**	**Y**	**Y**
**4. Woodhead et al., 2021**	**Y**	**Y**	**?**	**Y**	**Y**	**N**	**Y**	**Y**	**Y**	**Y**

## Discussion

This study is the first systematic review, to the best of our knowledge, to synthesize evidence on acceptance and access to COVID-19 vaccines among ethnic minorities and migrants. Most of the included studies were quantitative and assessed the acceptance of COVID-19 vaccines among ethnic minorities rather than migrants. There is consistent evidence that Black ethnic minorities in the UK and US report higher vaccine hesitancy than their White counterparts but, in the US, the picture with respect to Hispanics/Latinx populations and their White counterparts varies. Most studies that included Asians in the US found higher COVID-19 vaccine acceptance compared to Whites. The few studies that looked at migrants found higher vaccine acceptance compared to the general population. However, there was evidence that migrants face access barriers, due to a host of factors, the commonest of which related to a lack of confidence in COVID-19 vaccines, particularly due to mistrust of governments and health systems as well as lack of information particularly due to poor communication.

Vaccine hesitancy among ethnic minorities and migrants constitutes another barrier to uptake, largely driven by mistrust in the healthcare system that is widespread with ethnic minority and migrant communities. This can be traced to previous unethical research practices such as the Tuskegee syphilis study (Dawson et al., 2020). Such practices have led to a legacy of mistrust among ethnic minorities in the US and UK. Beyond these historical factors, ethnic minorities often face racial discrimination, which shapes their perspective on the health and wider governance systems, and migrants often face hostile health care environments due to a range of policies which aim to deter unofficial immigration by making life for those who are undocumented untenable.

Systemic racism and structural injustice are increasingly being recognized as a determinant of health (Bajaj and Stanford, 2021). This is exemplified by the response to the killing of unarmed Black people in the US which sparked protests in many cities across the country and in over 60 countries globally (Times, 2021). It is not inconceivable that the ensuing racial tensions coupled with racially divisive rhetoric by the then US president (CNNPolitics, 2020) might have further deepened mistrust and exacerbated vaccine hesitancy among ethnic minorities and migrants in the months preceding the vaccine roll out. The plausibility of such an association is supported by evidence that police shootings of unarmed Black Americans can have a demonstrable impact on mental health of the Black population in the same state (Bor et al., 2018).

A silver lining in our finding was that building relationships with ethnic/racial minority and migrant communities using local ambassadors improves acceptance of COVID-19 vaccines. This offers an approach to engagement with community and religious leaders to build trust, tackle misinformation and improve vaccine acceptance. An equally encouraging finding was that healthcare workers remained the most trusted source of information among ethnic minority and migrant groups. Such trust, if properly leveraged, could improve uptake among ethnic minorities and migrants. Health workers especially those from migrant and ethnic minority backgrounds themselves, can play a crucial role in improving trust, the importance of which was shown by Quinn et al. who found that a lack of diversity in clinical settings was associated with increased perception of racial discrimination and lower influenza vaccine uptake among ethnic minorities (Quinn and Andrasik, 2021). This is further supported by a study that suggested increasing numbers of Black physicians may result in improved quality of care for Black patients, perhaps by addressing conscious and unconscious biases (Alsan et al., 2022). An example of an approach which leveraged on health-workers’ racial diversity and trust to improve COVID-19 uptake among ethnic minorities is Loma Linda University's mobile vaccination clinic (Abdul-Mutakabbir et al., 2021). This approach, which included Black faith leaders and Black healthcare professionals with a vaccination clinic hosted in a church parking area improved vaccination uptake among Black Americans. By building a reputation of trust based on mutual partnerships, governments and health institutions can expect to improve vaccine confidence and uptake among ethnic minorities and migrants.

The detrimental effect of online misinformation has been highlighted even before the current pandemic (Larson, 2018). In what is being now being termed an “infodemic”, the spread of COVID-related misinformation is now widely acknowledged as a threat to the global efforts towards ending the pandemic (Zarocostas, 2020). The infodemic threat is even more potent given the evidence that anti-vaxxers are employing bots and trolls to drive COVID-19 vaccine hesitancy on social media (Hernandez et al., 2021). Equally worrisome is what Hernandez and colleagues referred to as “Health Care Provider Social Media Hesitancy”, a phrase denoting medical communities’ lack of engagement with social media (Hernandez et al., 2021). The authors reported that only 10% of the 1 million COVID-19 informational tweets they analyzed were from medical professionals. The authors further called upon healthcare professionals to “engage more in social media to counter the mounting vaccine-related infodemic”. Although Twitter and Facebook have employed measures to restrict the spread of “Fake news”, other platforms such as Telegram and WhatsApp still offer an outlet for propagation of COVID-19 disinformation. This calls for a more robust strategy to counter disinformation while taking steps to increase vaccine confidence among ethnic minorities and migrants.

Despite reassurances by most governments that neither identification nor payments were required to obtain COVID-19 vaccinations, undocumented migrants still remained distrustful of host authorities and healthcare systems. This skepticism is often understandable given the experiences of migrants with authorities in their countries of origin, in transit, and in their new homes, in some cases manifestations of an explicit policy to create a “hostile environment”, (Legido-Quigley et al., 2019) an approach that is contrary to commitments by governments to Universal Health Care set out in the Sustainable Development Goals and elsewhere (Forman et al., 2016). This poses a challenge for those offering support to migrants, in health care providers or in civil society, as they may need to challenge overzealous immigration authorities and, in doing so, may face attacks from xenophobic politicians and commentators in some sections of the media. The support they offer can take many forms, including provision of safe spaces where migrants know that their information will not be passed on, such as the Safe Surgeries Network organized by Doctors of the World in the UK (Safe surgeries network - Doctors of the World, 2022) to legal support, with a growing body of evidence on the contribution of strategic litigation in support of minority rights (Ezer et al., 2018). Furthermore, the messaging should state in clear terms that no of cost of services are involved with COVID-19 vaccinations.

There may be scope for explanation of the vaccine development process by religious and community leaders but this must recognize the challenges involved in overcoming past and current experiences.

A one size fits all approach risks excluding ethnic minorities and migrants. There is a need for culturally sensitive and context specific COVID-19 vaccine information that reflects their situation and beliefs, while reaching out to appropriate settings such as places of worship and community centers.

A key strength of our study lies in the fact that we assessed both demand-side barriers such as vaccine hesitancy and supply-side barriers such as vaccine access among two socially disadvantaged populations globally. Additionally, by including both quantitative and qualitative studies, we were able synthesize evidence on the extent of barriers to COVID-19 vaccination uptake as well its drivers. However, despite it being broad in scope, the review still had some weaknesses and limitations.

First, as noted above, we recognized the potential problems arising from the terminology employed-“ethnic group”. However, we were reassured that our search strategy was sufficiently sensitive, capturing papers that used many different terms, such as “racial groups/ minorities” and “Blacks”. Second, the methods and quality of the included studies varied substantially. Few studies focused primarily on ethnic minorities or migrants so these groups comprised a small share of the overall sample. The studies varied greatly in size, from 90 to 32,000, thus limiting the comparability of findings. Third, while some studies adjusted for possible confounders, others did not. Additionally, some cross-sectional studies had no comparison group. The qualitative studies employed snowball or purposive sampling which has an inherent risk of selection bias. This is understandable in studies of migrants given that many, especially those who are undocumented, are often difficult to reach. Fourth, the operationalization of acceptance varied across the studies. For example, some studies such as that by Gatwood et al. assessed vaccine hesitancy using a validated Vaccine Hesitancy Measurement Scale while others did not. Furthermore, studies such as that by Malik et al. assessed acceptance using 5-Likert scale survey instruments that dichotomized responses into “acceptance and non-acceptance” while other studies such as Kuhn et al. and William et al. dichotomized responses into “hesitant and not hesitant”. This heterogeneity in outcomes makes meaningful comparison difficult. Fifth, the majority of the studies assessed vaccination intentions. While there is some evidence that intentions mirror uptake trends, there is need for cautious interpretation as the decision to accept a vaccine is a result of complex interactions and influences that could change with time. Sixth, while we did not apply language restrictions, our search did not return eligible publications in any language other than English. This limits the generalizability of our findings. Although we have ensured fidelity to the strategy as set out in our PROSPERO submission, in response to a comment by a reviewer, we subsequently widened our search to include African Index Medicus and LILACS database but found no additional papers. Finally, we did not search for gray literature, nor did we consider pre-print publications. Including these types of publications could have yielded additional relevant results but at the risk of being misled, a particular problem in a field where there is so much disinformation.

Reasons behind low vaccine acceptance among ethnic minorities are plausible and in keeping with previous vaccination uptake patterns (Harrington et al., 2021). However, there is the need for more qualitative studies to provide much deeper understanding of how these factors affect vaccine uptake among ethnic minorities and migrants. Such qualitative studies could be used to interpret subjective experiences of ethnic minorities and migrants to define strategies that will ensure equitable access and delivery of COVID-19 vaccines.

Only a few studies reported on migrants’ access to and acceptance of COVID-19 vaccines, which is disappointing given estimated that there were over 270 million migrants in 2019 (The number of international migrants reaches 272 million 2022). Consequently, caution should be applied when drawing conclusions from our findings on migrants. The heterogeneity of the populations and the methods employed limit the generalizability of these studies. There is therefore the need for more studies of migrant's access to COVID-19 vaccines. This is necessary due to the limited number of studies and the different contexts in which they were conducted.

## Conclusion

There is consistent evidence that Black/Afro-Caribbean groups in the US and UK experience barriers to COVID-19 vaccine uptake associated with hesitancy. Conversely, the picture with regard to Hispanic/Latino populations in the US and Asian populations is mixed and likely context dependent. Most studies that included Asians in the US found high COVID-19 vaccine acceptance. Only a few studies reported on migrants, finding higher acceptance than in the general population. However, both ethnic minorities and migrants face many barriers to being vaccinated for a host of reasons, varying from poor communication to mistrust of governments and health systems. This points to a need to improve communication and transparency about vaccine development, working with religious and community leaders. However, there will be many challenges given the pervasiveness of racism experienced by minority groups, hostile policy environments faced by migrants and consequent distrust of authorities and healthcare systems. The experience with COVID-19 vaccination is consistent with that previously for other vaccines. However, despite the high health and economic toll of the pandemic on these groups, the amount of research that exists to understand their situation and address the challenges they face is inadequate. As a result, conclusions should be drawn with caution and care taken not to generalize, although this should not be an excuse for inaction as there are many obvious things that can be done to address the multiple barriers to accessing health services that these groups face. Meanwhile, there is a clear need for more qualitative research that can provide the nuance to inform the development of targeted interventions designed in full partnership with these communities.

## Declaration of Competing Interest

The authors declare that they have no known competing financial interests or personal relationships that could have appeared to influence the work reported in this paper.

## Appendix Supplementary materials

Image, application 1

Image, application 2

Image, application 3
