# Supplementary material for: Ethnic/racial minorities’ and migrants’ access to COVID-19 vaccines: A systematic review of barriers and facilitators
Source: J Migr Health. 2022 Feb 18;5:100086. doi: 10.1016/j.jmh.2022.100086 (PMC8855618; doi:10.1016/j.jmh.2022.100086)
Supplement: Supplementary file 1 [file mmc1.docx]

**Appendix 1. Full verbatim search strategy for PubMed**

("refugees"[MeSH Terms] OR "refugees"[All Fields] OR ("internally"[All Fields] AND "displaced"[All Fields] AND "person"[All Fields]) OR "internally displaced person"[All Fields] OR ("emigrants and immigrants"[MeSH Terms] OR ("emigrants"[All Fields] AND "immigrants"[All Fields]) OR "emigrants and immigrants"[All Fields] OR "emigrant"[All Fields] OR "emigrants"[All Fields] OR "emigrate"[All Fields] OR "emigrated"[All Fields] OR "emigrates"[All Fields] OR "emigrating"[All Fields] OR "emigration and immigration"[MeSH Terms] OR ("emigration"[All Fields] AND "immigration"[All Fields]) OR "emigration and immigration"[All Fields] OR "emigration"[All Fields] OR "emigrations"[All Fields] OR "emigres"[All Fields]) OR ("refugees"[MeSH Terms] OR "refugees"[All Fields] OR ("asylum"[All Fields] AND "seeker"[All Fields]) OR "asylum seeker"[All Fields]) OR ("refugee s"[All Fields] OR "refugees"[MeSH Terms] OR "refugees"[All Fields] OR "refugee"[All Fields]) OR ("migrant s"[All Fields] OR "transients and migrants"[MeSH Terms] OR ("transients"[All Fields] AND "migrants"[All Fields]) OR "transients and migrants"[All Fields] OR "migrant"[All Fields] OR "migrants"[All Fields]) OR "Ethnic Groups"[MeSH Terms]) AND (("covid 19"[All Fields] OR "covid 19"[MeSH Terms] OR "covid 19 vaccines"[All Fields] OR "covid 19 vaccines"[MeSH Terms] OR "covid 19 serotherapy"[All Fields] OR "covid 19 serotherapy"[Supplementary Concept] OR "covid 19 nucleic acid testing"[All Fields] OR "covid 19 nucleic acid testing"[MeSH Terms] OR "covid 19 serological testing"[All Fields] OR "covid 19 serological testing"[MeSH Terms] OR "covid 19 testing"[All Fields] OR "covid 19 testing"[MeSH Terms] OR "sars cov 2"[All Fields] OR "sars cov 2"[MeSH Terms] OR "severe acute respiratory syndrome coronavirus 2"[All Fields] OR "ncov"[All Fields] OR "2019 ncov"[All Fields] OR (("coronavirus"[MeSH Terms] OR "coronavirus"[All Fields] OR "cov"[All Fields]) AND 2019/11/01:3000/12/31[Date - Publication])) AND ("vaccin"[Supplementary Concept] OR "vaccin"[All Fields] OR "vaccination"[MeSH Terms] OR "vaccination"[All Fields] OR "vaccinable"[All Fields] OR "vaccinal"[All Fields] OR "vaccinate"[All Fields] OR "vaccinated"[All Fields] OR "vaccinates"[All Fields] OR "vaccinating"[All Fields] OR "vaccinations"[All Fields] OR "vaccination s"[All Fields] OR "vaccinator"[All Fields] OR "vaccinators"[All Fields] OR "vaccine s"[All Fields] OR "vaccined"[All Fields] OR "vaccines"[MeSH Terms] OR "vaccines"[All Fields] OR "vaccine"[All Fields] OR "vaccins"[All Fields])
